# Treatment Trials in Disorders of Consciousness: Challenges and Future Directions

**DOI:** 10.3390/brainsci12050569

**Published:** 2022-04-28

**Authors:** Michael H. Marino, John Whyte

**Affiliations:** 1 MossRehab Hospital, Elkins Park, PA 19027, USA; jwhyte@einstein.edu; 2 Moss Rehabilitation Research Institute, Elkins Park, PA 19027, USA

**Keywords:** disorders of consciousness, clinical trials

## Abstract

The evidence base supporting treatment interventions for patients with disorders of consciousness is limited, and rigorous treatment trials are needed to guide future management of this complex patient population. There are many potential study designs that can be employed to develop this evidence, but the process of selecting the optimal study design is challenging. This article reviews common obstacles that impede research progress in this population and a range of study designs that may be employed. In addition, we consider how the particular practical and scientific obstacles may drive selection of the optimal design and, in particular, how the optimal design changes as treatment research proceeds along the translational continuum from mechanistic discovery to real-world clinical impact.

## 1. Introduction

Treatment interventions for patients with disorders of consciousness (DOC) can have multiple goals. Early after injury, an intervention may be pursued in the hope of achieving more rapid global recovery, including of consciousness, without necessarily affecting the long-term outcome (i.e., shorter interval to final outcome). Amantadine HCl for individuals with traumatic DOCs falls in this category; it has been shown to accelerate recovery but may or may not affect the ultimate outcome [1]. Alternatively, a treatment might be intended to enhance the long-term outcome, without necessarily affecting the pace of recovery (i.e., longer duration of active recovery). In addition, treatments may be pursued in the subacute or chronic phase, to enhance performance in some way while under treatment, without necessarily affecting recovery or outcome in the absence of the treatment (i.e., symptomatic improvement). Zolpidem falls in this category for some patients. Its use can temporarily restore consciousness in the chronic phase, but the DOC returns when the medication wears off [2]. The goals of the treatment intervention will inform study design since, for example, crossover designs might be considered for symptomatic treatments but are logically inconsistent with treatments intended to alter the pace or level of long-term outcome. These goals will also shape participant selection toward those with more acute or more chronic injuries and other characteristics. We should also note that there is a wide range of treatments that are provided to maintain or enhance the physical health of patients with DOC, in hope that this will support a more positive functional recovery. In the text that follows, we will not address trials of these types of treatments. This text can serve as a practical guide for experimental treatment trials in disorders of consciousness and will also elucidate how a major paradigm shift must occur in how health care is delivered to DOC patients.

Study design is also closely related to the nature of the treatment and its mechanism of action (MOA). The field of rehabilitation employs treatments that rely on a wide range of principles of change from altering brain cellular processes to acquiring adaptive skills for coping with residual disability. However, when designing treatment interventions for patients who are vegetative or for many who are minimally conscious, the classes of treatment narrow to those that can be administered passively to alter brain function. These consist, at the present time, of pharmacologic treatments, electromagnetic treatments, and ultrasound. In principle, pharmacologic agents could address any of the three broad treatment goals described above. We have examples already of drugs that alter pace of recovery and provide temporary symptomatic benefit, as noted. To date, no neuroprotective agent given early has been demonstrated to alter long-term function after severe TBI but an effective agent in this class would be an example of the second category above—enhancing long-term outcome [3]. The evidence to date on electromagnetic and ultrasound treatments is insufficient to determine whether they have the ability to alter long-term outcome; to date, they have been studied primarily for their relatively short-term behavioral effects [3,4].

When patients reach the upper levels of the minimally conscious state, they begin to acquire the capacity to learn strategies and adaptations. Such active, learning-based treatment interventions take on greater prominence during recovery. Since trials of, for example alternative and augmentative communication systems (such as eye gaze for yes/no responses or brain computer interface devices), might rely on preserved language capacity and some form of motor signal for response, subject selection for such studies may be more appropriately designed around cognitive and behavioral capacities rather than specific features of brain biology.

**Figure 1:**
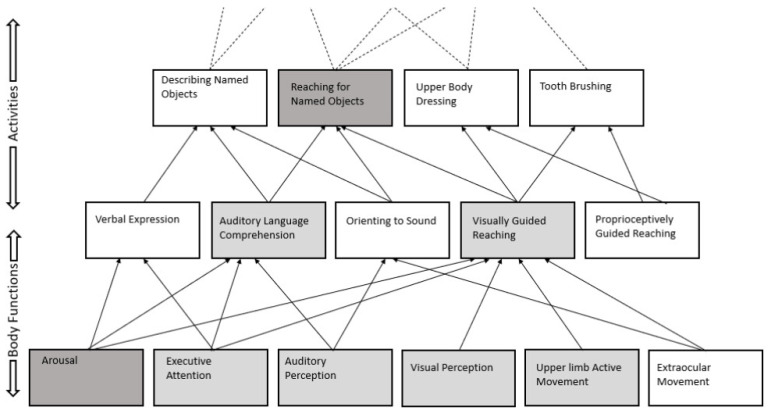
Enablement Model of MCS Functional Abilities.

Treatments for patients with DOC generally have an immediate mechanistic goal (e.g., to enhance arousal) and a more distal functional goal (e.g., to enhance reaching for objects during independent self-care as a result of greater arousal). It is critically important to recognize that these two goals rely on very different theoretical foundations. The claim that a given treatment enhances arousal as measured in a certain way is a statement of a *treatment theory*: that a particular set of active ingredients (drug, electrical modulation, etc.) acts through some mechanism of action to increase a measurable aspect of arousal [5]. In contrast, the prediction that an increase in arousal will lead to improved reaching during ADLs rests on *enablement theory*, a set of propositions of how change in one functional entity (i.e., arousal) produces change in other functional entities (i.e., reaching for objects) [5]. Importantly, the study treatment has no direct mechanism of action on “reaching for objects”, and whether an increase in arousal produces that change for a particular patient will depend on many other factors such as that patient’s motor skills and visual perception (See Figure 1). Thus, a clinical trial of a drug might very well be effective when measured with an outcome of arousal but ineffective when measured with an outcome of increased independence. Treatment theories propose tools to change specific clinical targets but are moot as to the remote functional impacts of those changes. In contrast, enablement theories predict the indirect downstream functional consequences of a change in one or more targets but are moot with respect to tools to directly change selected targets. Consequently, investigators pursuing early mechanistic research to support the treatment’s mechanism of action can concern themselves only with treatment theory. However, translational researchers seeking to establish the practical clinical impact of the treatment must also rely on enablement theory to predict which patients can benefit clinically from the specific individual treatment and/or what combination of treatments can target the constellation of impairments needed to result in meaningful functional improvement.

Figure 1 presents a partial enablement model of early MCS functional abilities in schematic form. The vertical axis represents increasingly complex functions as defined by the ICF, with basic body functions at the bottom, combining into simple and somewhat more complex activities in the upper rows. The arrows indicate the causal importance of multiple lower functional abilities on each higher one, with dotted lines at the top indicating causal influences on still more complex activities and aspects of participation. Enablement theory is concerned with the relative “causal weights” of these arrows. For example, an impairment in auditory perception might have a more profound effect on auditory language comprehension than on verbal expression. Visual perception and extraocular movements may both contribute to visually guided reaching, but perhaps visual perception is more critical (i.e., has a higher weight). Importantly, treatments that are successful in enhancing functioning as represented by one of these boxes (e.g., dark-shaded “arousal”) may not have an impact at higher levels (e.g., dark-shaded “reaching for named objects”) because of additional untreated functions (e.g., the several light-shaded functions) that are necessary for the functional performance in question, and which may, depending on the patient, also be impaired.

This complexity maps onto another important dimension in treatment research: the translational evolution of the research from early proof-of-principle studies to later clinical efficacy and effectiveness research. Early in the translational process, one will be primarily interested in testing the treatment theory and verifying that the hypothesized MOA for the treatment appears supported by the research. Thus, outcome measures closer to arousal, in the above example, would be appropriate. However, these are not meaningful measures of clinical impact or value. Accordingly, as the translational research process moves toward addressing real-world clinical benefit, one must seek dependent measures that are more functionally relevant [6]. However, along with this comes the above complexity: of all the patients who have the capacity to respond to the treatment’s MOA, many will not be able to demonstrate functional benefit because of the complex enablement links between arousal and those meaningful clinical outcomes. This issue cannot be solved by pursuing stronger treatments for arousal. Rather, one must select research participants for whom a change in arousal is predicted to lead to major changes in function (i.e., because the participants do not have additional confounding deficits) or participants who can be treated with the study agent along with several other treatments that will, collectively, result in meaningful functional changes. In either case, the researcher must be sophisticated not only about the treatment’s effects, but about the complex functional links embodied by enablement theory [7,8]. That is, the researcher must understand the task requirements of important areas of functional performance, in order to predict whether treatment of the selected target is or is not likely to translate into improvements in more complex capacities and activities.

## 2. Patient Selection

Patient selection is a critical component of study design. The investigator needs to determine which patients should be included and excluded from the study. A major challenge in the DOC population is the heterogeneity of the population itself, including the pathology of the underlying injury. DOCs can be caused by traumatic, vascular, anoxic, toxic, or hypoglycemic events, among others. Some areas of the brain are more susceptible to certain mechanisms of injury. For example, traumatic injuries are more likely to cause injury in the inferior frontal lobes and anterior temporal lobes [9]. Anoxic injuries are more likely to injure the watershed cortex, basal ganglia, cerebellum and hippocampus.

Even among patients with similar etiologies of injury, there is great heterogeneity. For instance, how similar is a patient with traumatic diffuse axonal injury with brainstem involvement to an individual with a large traumatic left sided subdural hemorrhage? Will their recovery trajectories be the same? They may both be vegetative or minimally conscious at some point, but how will their underlying injuries influence their recovery and their ability to demonstrate consciousness? How will it influence their response to a potential treatment?

Despite this large degree of heterogeneity of the population, there must still be overlap of injured structures or systems that are implicated in the control of consciousness, though the precise role of various neural structures in the regulation of consciousness remains controversial. Our current conceptual framework of DOC relies primarily on phenotypes (observable behaviors manifested by motor response and overt evidence of cognition) and their relation to underlying pathology. For example, it has been demonstrated that patients with coma of any etiology will have injury and deficits related to the arousal system mediated by the reticular activating system. Patients in a vegetative state have recovered basic function of the arousal system but are likely to have thalamic injury [10]. Patients in the minimally conscious state are less likely to have severe thalamic injury, but are more likely to have injury to the thalamocortical projections underlying higher levels of cognitive processing [11].

While the injured systems may share some overlap across etiologies, the mechanisms underlying the potential for recovery and for treatment response may still vary. Depending on etiology and severity, some crucial systems may be damaged beyond natural repair or so profoundly that they are incapable of responding to certain treatments. We must also consider the possibility that differences in patient response may be related to whether the damage has occurred to primary computational circuitry (e.g., transforming auditory signals into recognizable language) or to modulatory systems that regulate function of one or more computational systems (e.g., ascending neurotransmitter systems that modulate signal-to-noise ratio), or both. This is analogous to how modulatory systems can act to inhibit or enhance the experience of pain from nociception [12]. Response to a neuromodulatory treatment will depend not only on the severity of damage to the modulatory system but on the residual integrity of the necessary processing circuitry.

This leaves the investigator with several decisions to make regarding the study population and criteria for inclusion or exclusion. One could proceed simply by limiting the study to all patients with a particular etiology of injury, such as including patients with traumatic injuries and excluding all other causes. Alternatively, the study population could include only patients who are minimally conscious, regardless of the etiology of injury and exclude all patients in the unresponsive wakefulness state. Or the study could target patients with large left hemispheric strokes only. The possibilities are dizzying, and in some cases they can feel arbitrary. As our field advances, we anticipate the further classification of DOC patients into endotypes, as endorsed by the Curing Coma Campaign. An endotype is a constellation of disease features stemming from a biological mechanism and is associated with a predictable clinical course and response to treatment. Identification of endotypes will alleviate the challenges of heterogeneity in this patient population by subgrouping clinically similar but biologically heterogenous patients into purer biological classes [13]. The patient endotypes selected for inclusion, then, must match the biological mechanism of the study treatment as discussed below.

We suggest an approach to patient selection that is primarily based on the foundation of treatment theory. The Rehabilitation Treatment Specification System (RTSS) is a method in which any rehabilitation treatment can be characterized utilizing the three elements of treatment theory: targets, ingredients, and mechanisms of action. This approach provides a framework that will guide researchers on how to systematically test of effects of the treatment ingredients on their targets [14]. Patient selection will start with an understanding of the active ingredients of the treatment, the biological target that they are hypothesized to modify, and the hypothesized mechanism by which those ingredients act to change the target. The investigator should consider how the mechanism of action is related to the etiology of injury. For example, etiology of injury (i.e., anoxic or traumatic) would be more relevant for a study of hyperbaric oxygen treatment in the acute or early post-acute phase than it would be in the chronic phase. The investigator should also consider whether the mechanism of action is related to the brain systems impaired, regardless of etiology. For example, we know that cognitive activity consistent with minimal consciousness requires activation of broad cortical networks dependent upon thalamocortical connectivity. Investigators studying deep brain stimulation of the thalamus to increase thalamocortical activity could consider applying their treatment to patients with any etiology of injury that resulted in particular patterns of thalamocortical damage. Alternatively, the investigator could consider whether the treatment’s mechanism of action is related to a behavioral capacity irrespective of the brain systems impaired. For example, if testing the efficacy of a simple augmentative communication system, one could consider selecting a sample of patients who have demonstrated some behaviors consistent with preservation of language (i.e., command following or verbalization) irrespective of the mechanism of injury, location of injury or systems known to be injured.

An ideal is to only enroll participants in a treatment trial who have the neural substrate necessary to respond to the treatment if the treatment theory is accurate. This approach, referred to as “sample enrichment,” ensures that failures to respond to the study treatment reflect inefficacy rather than selection of “untreatable” patients. In the above communication example, if the study contained individuals with global aphasia, this might make it appear that an augmentative communication system was ineffective, whereas if it was studied only in patients with preserved language but motor output limitations, a different conclusion might have been reached. Of course, the necessary neural substrate is the hypothesized treatment target. As such, a negative treatment trial in an enriched sample will refute the hypothesis about the treatment’s effect on the hypothesized substrate; a subgroup response will suggest the need to further refine the definition of the necessary neural substrate. Thus, whether positive or negative, treatment trials conducted in well-specified patients with well-specified treatments can refine the treatment theory over time.

## 3. Specifying Experimental Treatment and the Comparison Condition

The next challenge in designing a clinical trial for patients with DOC involves specifying the experimental treatment and its corresponding comparison condition, or control. As previously stated, patients with DOC are a heterogenous population and as such, their range of outcomes is also varied. They can start as non-participatory in the rehabilitation process, only receptive to passive interventions, and then progress to partially participatory. Some even progress to fully participatory with conventional rehabilitation programs and treatments. In the following discussion, we will focus most heavily on treatments that can be delivered passively to unconscious or minimally conscious patients. As consciousness improves, the range of rehabilitation treatments of interest increasingly overlaps with those delivered to individuals without DOC.

The range of experimental treatments to consider in the field of DOC rehabilitation is also broad, including pharmacologic agents, electrical and magnetic neuromodulatory treatments, passive exposure treatments (e.g., sensory stimulation), as well as complex multidisciplinary rehabilitation programs that combine such treatments with a range of medical and physical management techniques. We recommend, once again, relying on the fundamentals of treatment theory to specify the active ingredient(s) in the treatment intervention, its hypothesized mechanism of action, and the brain system(s) that are directly altered by the treatment. For most pharmacologic agents, the drug itself is the active ingredient with a well understood or hypothesized mechanism of action in most cases. In studies of pharmacologic agents, the control condition frequently involves use of a placebo. A placebo should be harmless, completely inert/inactive, and should closely resemble the active treatment [15,16]. Inert placebo pharmacologic agents are readily available. The ethics of placebo administration should always be considered, as it may be unethical to deny a patient an established treatment. In the field of DOC, the only firmly established pharmacologic treatment is the use of amantadine to promote neurologic recovery in patients with traumatic injuries between 4 weeks and 16 weeks post-injury [1]. A culture of frequent off-label use of pharmacologic agents, however, may reduce the acceptability of placebo-controlled trials in the eyes of caregivers and clinicians even if the evidence supporting off-label use is sparse.

Electromagnetic and physical forms of neuromodulation are also areas of treatment research in DOC, including transcranial magnetic stimulation (TMS), transcranial direct current stimulation (tDCS), peripheral nerve stimulation, pulsed ultrasound, and others [3]. The active ingredient for these neuromodulatory treatments is a form of physical energy delivered to an appropriately sensitive neural structure. Creating a “placebo control” for studies of this kind presents a challenge. Sham devices can be used but they have limitations, particularly with patients who have some awareness. Some medical devices create a sensation that is experienced by the patient and may be hard to reproduce. In some cases, it is unclear whether the sensation created is actually an active ingredient. Even if the sensation is not an active treatment ingredient, it may promote a strong placebo effect. As a result, the number needed to treat to detect a meaningful difference between active and sham treatment may be larger than the number needed to treat in clinical practice. If the sham device does not reproduce the sensation of treatment, it may unblind a conscious patient. The use of sham devices also complicates blinding of the provider as they may recognize the device as sham and not active treatment. In those cases, it becomes more critical to blind the individual who is measuring outcome to protect the internal validity of the study [17].

Studying the effectiveness of a complex intervention such as a multidisciplinary DOC rehabilitation program presents a much greater challenge. Rehabilitation programs tend to be interdisciplinary, multifaceted, and are designed to have effects on multiple targets or goals [16]. Although each patient with a DOC may be treated in “the same” complex rehabilitation program, the set of specific treatments received within that program will likely be unique to each patient. (Patients with hypertonia will receive treatment for it while those without hypertonia will not, etc.) Currently, complex multidisciplinary programs are composed of a poorly specified collection of treatments addressing a variable set of treatment targets with the goal that the aggregate effect of all these specific treatments will translate into improvements in larger treatment aims, such as “functional independence”. However, no individual treatment provided has a target of “functional independence”, so tracing the treatment ingredients that produced this aggregate effect is challenging. At present, we recommend focusing on studies of specific treatment targets related to consciousness, with well-defined targets and well-specified treatments. Separately, we can study the impact of complex treatment systems that attempt to incorporate as many evidence-based treatments as possible, while focusing on more macro outcomes such as functional independence. Over time, the proportion of focused treatments of proven efficacy that are incorporated into such complex programs should increase and the number of ineffective treatments be reduced, such that the effectiveness of the complex programs on macro aims should increase.

Developing control conditions for trials of complex rehabilitation interventions also is a challenge. No true placebo—intended to control for known and unknown biases—exists for non-pharmacologic and complex rehabilitation interventions. Instead, one must consider what specific sources of potential bias require control (i.e., natural recovery over time, practice effect from repeated use of outcome measures, investigator bias, etc.), and design one or more specific controls in response. The simplest control method is the no-treatment or wait-list control group. This group is assessed the same as the treatment group but receives no treatment. This controls for the effects of time/recovery and of repeated testing. However, prolonged non-treatment may be ethically unacceptable and, even if not unethical, may be undesirable to patients or caregivers. A waitlist control (where outcomes are measured in parallel to the treatment group but treatment is delayed until after the post-treatment assessment) may be more acceptable than a no-treatment control but during periods of acute recovery, the waiting period may put participants on different slopes of the recovery curve, as well as being unacceptable to caregivers [15,16,17,18].

Another strategy for the control condition for complex interventions involves creating a placebo analogue condition. These are control conditions that serve as analogues to a medical placebo. This involves designing a plausible treatment that is irrelevant to the true treatment target. These sham treatments, also referred to as pseudo treatments or spurious treatments, can control for the effects professional time and interaction, social contact, and cognitive stimulation. Unfortunately, they can add to the cost of the trial as two separate protocols are needed and can take more time to complete [15,16]. Moreover, it is often challenging to design a treatment that is both plausible and inert.

If withholding any active treatment is inadvisable on ethical grounds or is not practical (studies with a no treatment control group may struggle to find willing enrollees), then the control condition can consist of usual care. This can be achieved by comparing a group receiving an experimental treatment to a group receiving usual care. Alternatively, the design could consist of a group receiving the experimental treatment plus usual care against a group receiving usual care only. It may be difficult to detect a treatment effect if usual care has some degree of efficacy and may require large numbers of enrolled patients. This can represent a significant limitation in rehabilitation and DOC research where numbers of enrolled patients are typically small. The major barrier to the use of usual-care control groups comes in defining what usual care consists of and standardizing it. Without that standardization, one may not be confident that usual care is lacking in the active ingredients under study. While it is unlikely that something like TMS is frequently delivered during usual care, a trial of sensory stimulation will have to grapple with how much stimulation is experienced during usual care. Besides being poorly defined, there is frequently no accepted standard in the delivery of most complex rehabilitation treatments [15,16]. This can bring into question its viability as a control condition. The options include attempts to rigorously structure and operationalize usual care to make it more homogenous. This will complicate experimental design and may run the risk of discouraging treaters who typically practice with more autonomy. Alternatively, the experimenter could mitigate these effects by using a multicenter design that would inherently involve treaters with different levels of skills and expertise and then perform multivariate analyses to adjust for these variables [17].

If there are two or more well-defined forms of complex treatment, one can also consider a comparative effectiveness trial in which patients are randomized to “Program A” or “Program B”, measured against a relatively macro outcome. This will provide empirical insight into which form of service delivery produces better results at the global level but will not easily identify the component treatments that contributed to that outcome.

Importantly, the appropriate control condition depends on the phase of research. Early phases of research are typically intended to support the treatment theory by demonstrating that specific ingredients are causally implicated in a clinical change. In this case, it is critical that the active ingredients of the intervention are reduced or absent in the comparison condition. Later stages of research are more related to enablement theory: determining which combinations of treatments in which kinds of patients lead to which global improvements. Here, the emphasis is less on understanding the mechanism by which each ingredient exerts change, and more on the aggregate clinical effect of some method of selecting and assigning multiple treatments to specific patients [2].

## 4. Selecting the Outcome Measure

The next clinical trial design challenge is selecting the correct primary outcome measure. Naturally, most researchers and clinicians will want to study and measure the outcomes that seem most important in this population such as recovery of consciousness or independent function. Measures of independence in mobility and activities of daily living, as well as long-term outcomes such as return to work or school have also been prioritized. Accordingly, a variety of well-established scales have been used to measure these outcomes, from the Coma Recovery Scale-Revised, which assesses return of consciousness, to the original Glasgow Outcome Scale (GOS), Glasgow Outcome Scale-Extended (GOS-E), or Disability Rating Scale, which assess global functioning, with a myriad of measures of mobility, cognition, ADLs, psychosocial functioning, and community participation in between [19,20,21]. Indeed, the plethora of available outcome measures has led the National Institutes of Health to promote the adoption of “Common Data Elements” (CDEs) to help structure the optimal selection of measures for brain injury research [22].

The frequent use and general acceptance of a measure’s validity does not mean that it is ideally suited for a given clinical trial. In fact, it has been suggested that the failure to demonstrate efficacy of acute neuroprotive treatments can be attributed to improper selection of outcome measures. Measures chosen may be insensitive to detect meaningful change, may be mismatched to the intervention being tested, or may be psychometrically flawed [23]. Any of these will lead to an increased chance of rejecting an effective treatment (type 2 error) [16].

Researchers should first consider where along the translational research pipeline the study is taking place and how that relates to principles of treatment theory and enablement theory. While traditional drug trial phases do not always directly correlate to rehabilitation research, they do provide a framework for comparison. Drug phases 1 through 3 are often similar to proof-of-concept studies and early efficacy studies in rehabilitation. Studies at these early phases seek initial evidence elucidating the underlying mechanisms of a treatment. The purpose is to demonstrate that the treatment can work in a defined population. These early phases of research should rely on treatment theory to determine outcome measures, since one first needs to establish that the treatment ingredients have the predicted effects on the treatment target; only if they do does it make sense to ask what practical clinical value these target changes may provide (enablement effects). Treatment theory should be used to identify the specific functional change anticipated to result directly from the treatment. Outcome measures can be derived from this target function [6,24].

When selecting outcome measures, it is helpful to bear in mind the World Health Organization’s International Classification of Functioning, Disability and Health (ICF) [25] for describing and measuring health and disability. Early stage research is frequently aimed at addressing body structure or function (e.g., minimizing neuropathology and enhancing processing in specific neural networks). This is useful for early trials because if a treatment is able to show improvement in its immediate target, it can be a candidate for further study of more downstream effects. For example, if a medication shows improvements in arousal in DOC patients as measured by eye opening, it may be a good candidate for further study to determine whether and in whom an improvement in arousal also leads to an improved level of consciousness or enhanced mobility. In this case, the level of consciousness would represent a downstream effect that is several steps removed from the target function of the earlier studies (i.e., arousal/eye opening). Conversely, if the medication fails to show the predicted improvement in arousal, this suggests it cannot have those positive downstream effects (at least by the predicted mechanism, and at least in the types of patients studied).

Later stages of research should build on the work carried out in the earlier stages but can then ultimately progress to addressing the ICF levels of activity and participation. Once the efficacy of the treatment target is established, the next step involves determining the clinical utility of the treatment in in terms of its functional benefits to particular kinds of patients [2]. For these types of later studies that are further along the translational research pipeline, such as effectiveness studies, enablement theory will guide the choice of outcome measure, as well as the selection of patients with the potential to experience that downstream effect. Returning to the arousal example, an earlier stage of research might have determined which kinds of patients have the neurocomputational substrate required for arousal to increase consciousness. Thus, the next stage might enroll only patients with that substrate (sample enrichment with a relevant endotype) and now test the effectiveness of the intervention in enhancing not merely arousal, but consciousness. The further downstream the treatment goal is (such as walking independently, or returning to work), the more it will be dependent on a myriad of factors outside the direct effect of a single treatment. Thus, tracing the effects of a treatment intervention on such distal outcomes requires either selecting a patient endotype (if such exists) where the treatment target is the primary obstacle to improving the distal outcome *or* enrolling patients with more heterogeneous endotypes into a system of care containing algorithms that match individual patient characteristics to individual treatments intended, in the aggregate, to improve the distal outcome [6].

## 5. Study Designs

When considering experimental design, the researcher must match the question being asked to its relevant phase in the translational research process. As we will see in this section, some study designs are more appropriate for earlier phases rather than later phases, and other designs vice versa. Any intervention that we study in health care may lead to an effect on the target that results in measurable change. Even if we cannot directly observe the causal chain linking the intervention to changes in the target, we can infer that causal relationship if the potential confounding causes are excluded by the design. How strongly we can trust that inference depends largely on the experimental design. Often there is no realistic way to exclude all possible confounds and then one must settle for a series of studies that separately attempt to control for different alternative hypotheses. It is the randomized controlled trial (RCT) that is considered the gold standard for strength of inference and accordingly, for strength of evidence. The RCT is designed to ensure that the measured results are due to the intervention and not due to any other variables. The classic design for an RCT is a parallel group design in which one group is subjected to an experimental intervention, and the other group (the control group) is subjected to placebo only [1]. Great effort is made to ensure the two groups are comparable in all the ways that would otherwise determine the outcome of interest, so the only thing differentiating them is the randomization process that divided them into experimental and control groups [26].

Review of any recent literature discussing evidence-based practice will reveal countless cries for more RCTs to guide clinical care. However, while the RCT is the standard bearer for internal validity, there are limitations to the RCT, many of which have been covered previously in this manuscript. There can be challenges in blinding administrators to the largely non-pharmacologic interventions common in rehabilitation. High costs and complexity in managing the RCT are common challenges, particularly when embedding multicenter trials in heterogeneous service systems [16,26]. Recruitment challenges abound for RCTs including frequent resistance to being assigned to a control group. Strict inclusion and exclusion criteria can limit numbers of participants and lead to lengthy delays in reaching the planned sample size. Additionally, most RCTs are conducted in specialized research settings or academic centers [24]. The relatively low prevalence of DOC patients in a given region combined with the difficulties in transporting individuals with typically severe disability further compound the challenges to enrollment in RCTs. Perhaps the greatest single limitation of RCTs is their lack of generalizability. Typical RCTs frequently involve near ideal conditions, with strict adherence to treatment by clinicians and participants, in a select patient population. Many RCTs are best suited for earlier phases of the translational research process, when establishing the active ingredients is more critical than understanding their practical impact. Effectiveness studies are conducted later in the translational research process and under more real-world conditions with heterogenous patients and clinicians in settings with varied levels of expertise [6].

As there exist many challenges to parallel group placebo-controlled trials, other between group experimental designs can be considered as viable alternatives. Adaptive designs, as the name implies, are dynamic in that they involve changing the sample size or dropping a treatment arm mid-trial based on analysis of the data acquired. The benefits of such designs include the ability to drop a treatment arm that has been determined to be futile while continuing to evaluate other more promising treatment arms. They can also adjust the sample size to obtain sufficient power to support a preliminary finding. Extensive pre-trial planning is a critical component of adaptive trials. Bamman et al. suggest the following key considerations when planning an adaptive design: (1) Determine who will examine the data. (2) Determine the criteria for how/when sample size will be increased. (3) Determine criteria for whether a treatment arm should be dropped and who will make that decision. (4) Closely consider the statistical implications of examining the data for type I error and ensure there is sufficient power to support the correct decision. (5) Consider the implications of dropping a treatment arm or increasing enrollment on participants, investigators, the trial sponsor, the safety monitoring board and the institutional review board [24].

The sequential, multiple assessment, randomized trial (SMART) is a design for chronic conditions in which there is great heterogeneity in treatment response among participants. In these designs, non-responders to the intervention are re-randomized into alternative treatment arms. Thus, this type of design may offer a benefit in contexts where prolonged randomization to a placebo group or ineffective treatment may lead to excessive study dropouts. Outcomes in SMART designs are generally dichotomized into treatment success or failure. We foresee utility in this type of design in the DOC population in identifying endotypes and identifying characteristics of responders and non-responders to a hierarchy of treatments. Powering a SMART design adequately is a challenge, particularly if there are several levels of treatment and re-randomization [24]. In addition, the impact of a given treatment may also vary by when in recovery it is assigned.

Randomized controlled studies can be dichotomized into explanatory RCTs and pragmatic RCTs. As discussed, explanatory RCTs are often conducted in the early phases of research and they seek to answer theoretical questions, elucidate treatment principals, or establish the mechanisms underlying treatments. Pragmatic RCTs are between group designs conducted to answer questions regarding whether an intervention is clinically effective, or whether it works better than an alternative treatment [26]. Building off of the core concept of a pragmatic randomized controlled trial is the practical, or pragmatic, controlled trial. Pragmatic controlled trials (PCTs) are useful in their ability to combine the rigor of an RCT while also providing data on real-world effectiveness. Califf and Sugarman proposed three key attributes of the PCT: (1) intent to inform decision makers, as opposed to elucidating a biological or social mechanism; (2) intent to enroll a study population that is representative of the patient/population and exists in clinical settings relevant to the decision in practice; (3a) intent to streamline procedures and data collection for the trial to focus on adequate power to inform clinical and/or policy decisions; (3b) intent to measure a broad range of outcomes. Califf and Sugarman go on to present a common-sense definition of PCTs as: “Designed for the primary purpose of informing decision makers regarding the comparative balance of benefits, burdens and risks of a biomedical or behavioral health intervention at the individual or population level.” Pragmatic controlled trials are best conducted at later phases of clinical research [27], and are better suited to treatments or treatment packages intended to have effects on macro outcomes of general importance, for the reasons discussed previously. PCTs can assess the risks, costs, and benefits of treatments in clinical practice (“comparative effectiveness research”). Because they use more diverse samples and are less focused on treatment adherence, they can provide valuable information on the real-world effectiveness of treatments. If well designed, PCTs can be conducted with strong internal as well as external validity [16].

Within-subject trials are experimental designs in which each participant is exposed in sequence to every experimental condition. The primary advantage of this design is that each subject serves as his/her own control. An example of a within-subject design is the crossover trial, where each participant is randomized to a different sequence of treatment (e.g., AB or BA) [28]. In any within-subject design, it is important to consider the effects of time and sequence since the pace of spontaneous recovery may decelerate between the first and second treatment phases [29]. In regard to time, a maturation or natural recovery process could take place that will potentially affect the results, as noted with the SMART design. In regard to sequence, the order of treatments applied could affect the outcome from a carry-over effect [16]. Thus, crossover trials are most viable when the pace of change of the outcome of interest is slow in the absence of treatment, where the effects of each treatment are fully reversible when the treatment is stopped, and where the onset and clearance of the treatment effect are reasonably fast.

The N-of-1 trial (or single-subject experimental design) is a unique variation of a within-subjects design. As the name implies, N-of-1 trials include only one participant subjected to an intervention with multiple repeated observations to determine the presence or absence of effect from the intervention. A typical format will involve a sequence of baseline measurements, followed by an intervention, then repeated measurement of outcome/effect post-intervention. To account for the confound of natural recovery over time, N-of-1 trials, like other crossover trials, are ideally conducted in chronic conditions and with interventions that are expected to have a rapid effect. For these reasons, chronic DOC patients would represent a reasonable target for N-of-1 trials. In the early post-acute phase of DOC, not only is natural recovery a confound, but the large variability in day-to-day performance can obscure the intervention’s effect. To strengthen the evidence of an N-of-1 trial treatment effect, a washout period with removal of the intervention could be considered, in which case a decrement in the outcome measure (depending on the nature of the treatment) may be expected. This type of design, referred to as a reversal design or ABA trial, works best if the intervention has a short washout period [24]. However, the investigator should bear in mind that a decrease in response following removal of an intervention/treatment should not occur if that intervention was expected to truly change the trajectory of recovery as opposed to temporarily elevating the patient’s performance. A clear limitation of the N-of-1 trial is that it establishes the efficacy of the intervention only in the individual studied and cannot provide generalized conclusions. Therefore, we suggest that investigators consider a series of N-of-1 trials in which all patients receive the same intervention, with later group analysis of clinical differences between treatment responders and non-responders [30]. While the push for evidence-based medicine has elevated the RCT to the most desirable study design, we acknowledge that there will be circumstances in which an RCT, or indeed, any prospective experimental design, is impractical. In these instances, we advocate for the use of propensity scores in observational trials. Propensity scores can be used to identify the factors that predict whether a patient will or will not receive the treatment being studied. These scores can then be used to group patients into cohorts with comparable probabilities of receiving the treatment, but then evaluate the effects of actually receiving the intervention or not within each group [31].

Even with all of these options for experimental design, the challenge remains on how to best bridge the efficacy of RCTs with the practical challenges of delivering health care to a heterogenous population in varied clinical settings delivered by clinicians with a broad spectrum of clinical expertise. First, the authors advocate for delivery of care to DOC patients in specialized programs that meet the minimum requirements for taking care of this unique population [32]. Second, we imagine a treatment approach in which the clinician draws upon a vast repository of efficacy studies and uses that knowledge to apply treatments to specific impairments identified in their patients. In this sense, we do not foresee the improvement in outcome in DOC patients as being solved by a single RCT demonstrating efficacy, but by the gradual accrual of more evidence-based treatment and the simultaneous growth of research on complex systems for optimally delivering those treatments.

## 6. Practical and Ethical Considerations

The choice of study design requires consideration not just of matters of internal and external validity but also issues of feasibility and clinician and caregiver acceptance. A design that is perfect on paper will not add to the evidence if it fails to obtain IRB approval or cannot fulfill its enrollment goals. Indeed, very often, practical and ethical considerations require compromising on the design that might otherwise be optimal. Where this results in the inability to eliminate all confounds, one may have to settle for a series of studies that attack those potential confounds sequentially.

Clinical trials in rehabilitation frequently suffer from low enrollment. This may be compounded when working with such a specialized patient population as those with DOC or even those with a particular endotype of DOC. There may be insufficient numbers of patients in any one setting, necessitating a more complex and expensive multicenter trial [26]. The researcher must consider the time point along the acuity spectrum at which patients are enrolled. Larger numbers of DOC patients are available for enrollment from the intensive care unit or acute care hospital. However, the heterogeneity of that population is high, with some patients anticipated to recover briskly regardless of treatment (and, thus, adding “recovery noise” to the placebo or comparison group). The patients enrolled at progressively later times post-injury will have inherently lower heterogeneity. Improved identification of endotypes, as championed by the Curing Coma Campaign, will have a marked effect on identifying appropriate patients for enrollment at each time point post-injury. Endotype specification will allow for the exclusion of patients who are either unlikely to respond to the intervention, or whose course of recovery will be too rapid to allow for adequate evaluation of the intervention.

Once the patients are identified, the next hurdle is identifying the appropriate decision maker to provide surrogate consent. In the absence of legal guardianship, common law establishes an order of legally authorized representatives (LARs) for surrogate decision making. For clinical decisions, a looser standard of “the most involved” family member may be followed, whereas research consent may demand stricter adherence to the legal order of family members, even if they are less involved or difficult to reach. In our experience in working collaboratively with other US investigators, we have found that laws vary from state to state regarding the authority of legal guardians to consent to research. The role of the legally authorized representative is to make the decisions the impaired person would make, but very few individuals have had discussions about their beliefs and desires regarding medical treatment and research in the context of unconsciousness. Even fewer have legal documents detailing their desires in that regard. Additionally, family members are frequently significantly emotionally distressed surrounding the time of hospitalization of their loved ones and can be hard to approach for research consent. This becomes exponentially more difficult if their loved one is medically unstable.

When planning an RCT, the researchers must always answer the question: Is it ethical? For example, is it ethical for a patient to be randomized into the placebo group and/or require prolonged placebo use? To address these ethical questions, the researcher must consider the concept of equipoise. Equipoise dictates that an honest null hypothesis exists regarding the difference between the treatments being tested. The tenets of equipoise can also be met if the research is justified by its potential clinical value and the study subjects’ health will not be adversely affected. In order to address these issues, the researcher must consider the current state of the evidence for the treatment being withheld. This must be compared to the potential value of the study. Even when the tenets of equipoise are met and the study has been deemed ethical by the relevant institutional review board, the study still has to be acceptable to the caregiver. Cultural issues, health beliefs, socioeconomic status, and mistrust of research are frequently cited barriers to enrollment, and this can be particularly true for minority populations [33]. Caregivers may have strong beliefs regarding the superiority of a particular treatment, even if the evidence for its use is lacking, and may be reluctant to enroll their loved one. Reluctance towards enrollment may be related to the study design itself. Decision makers may be reluctant to have the patient enroll due to fear of being randomized into a placebo arm or no-treatment control group, even when there are no evidence-based alternative treatments available [26,34]. Once randomization has occurred, caregivers of patients in either treatment group may seek treatment outside of the study, particularly if the study intervention period is long or their family member is doing poorly, introducing treatment confounds or resulting in biased attrition [26,34,35].

Another source of attrition is the medical complexity of this patient population. Patients with DOC suffer from a high degree of medical complications, sometimes leading to medical instability that can result in withdrawal from treatment trials. Known common medical complications in DOC patients include hypertonia/spasticity, agitation/aggression, urinary tract infections, pulmonary infections, sleep wake cycle disturbances, neurogenic bowel, paroxysmal sympathetic hyperactivity, seizures, and late intracranial hemorrhage [36,37]. Thus, treatment trials must look for ways to select patients less likely to experience disqualifying medical complications, and/or consider analysis strategies that are robust to these treatment dropouts.

It is anticipated that patients with DOC will require a proxy decision maker or legally authorized representative. However, we need to anticipate that advances in neuroimaging technology will likely lead to improved communication in patients with DOC. Peterson et al., explore the ethical considerations of informed consent in DOC patients who can communication using yes/no responses detectable only on advanced neuroimaging. While acknowledging that only a small percentage of DOC patients will have the cognitive ability to communicate in this way, they foresee this communication medium becoming more accessible and common as its availability increases. They propose a novel process for involving patients in medical decision making for clinical trials utilizing a clinical vignette presented to the patient that they demonstrate understanding of by answering yes/no questions. This is then augmented by the use of supported decision making in which a trusted family member or friend assists the patient in exercising their decisions [38].

In 2022, Young et al. published an in-depth article exploring the ethical considerations in disorders of consciousness. They identify four central categories of ethical challenges in clinical trials of DOC: (1) autonomy, respect for persons and consent of individuals who lack decision-making capacity; (2) balancing unknown treatment benefits and risks; (3) disclosure of investigational results pertaining to consciousness; and (4) equitable access to clinical trials to minorities. They also address disclosure of investigational results pertaining to consciousness [33].

## 7. Conclusions

In this paper, we have strived to provide practical guidance on navigating the challenges of designing and implementing clinical trials for patients with DOC in our current medical research environment. However, we believe that in order to truly advance the field of DOC research, a major paradigm shift must occur in how health care is delivered to these patients and how institutions interact with each other. This paradigm shift was described in The Mohonk Report, which concludes that the “structure of the existing healthcare system does not adequately address the complexity of problems that impact patients with very severe brain injury and their families. This situation negatively impacts the quality of clinical care and constrains much needed research in this area” [39]. The report proposed a radical restructuring of health care institutions existing within a network. The proposed network includes specialized research institutions linked to academic medical centers with access to research technology and expertise in research design. These are referred to as Centers of Excellence (CORE). The next level of the network is comprised of acute rehabilitation centers (ARC) with expertise in DOC rehabilitation. The last level in the network comprises skilled nursing facilities (SNF), each one linked to an ARC. Funding from government bodies supporting clinical care and research would facilitate maintenance of large data sets for longitudinal data collection, transfer of patients among the three levels of the network for clinical and research purposes and consolidation of resources for identifying surrogate decision makers. Several of the ideas from the Mohonk Report, such as maintaining large data sets, development of proof-of-concept clinical trials, and strengthening prognostication and long-term recovery have been reinforced by the Curing Coma Campaign [13].

## Data Availability

Not applicable.
